# Hybrid Materials Based on the Embedding of Organically Modified Transition Metal Oxoclusters or Polyoxometalates into Polymers for Functional Applications: A Review

**DOI:** 10.3390/ma7053956

**Published:** 2014-05-20

**Authors:** Mauro Carraro, Silvia Gross

**Affiliations:** 1 Dipartimento di Scienze Chimiche, Università degli Studi di Padova, via Marzolo 1, I-35131 Padova, Italy; 2 ITM-CNR, UOS di Padova, via Marzolo 1, I-35131 Padova, Italy; 3 Istituto per l’Energetica e le Interfasi, IENI-CNR and INSTM, UdR Padova, via Marzolo 1, I-35131 Padova, Italy

**Keywords:** oxoclusters, polyoxometalates, organic-inorganic hybrid materials, polymers, functional properties, POM, transition metals

## Abstract

The covalent incorporation of inorganic building blocks into a polymer matrix to obtain stable and robust materials is a widely used concept in the field of organic-inorganic hybrid materials, and encompasses the use of different inorganic systems including (but not limited to) nanoparticles, mono- and polynuclear metal complexes and clusters, polyhedral oligomeric silsesquioxanes (POSS), polyoxometalates (POM), layered inorganic systems, inorganic fibers, and whiskers. In this paper, we will review the use of two particular kinds of structurally well-defined inorganic building blocks, namely transition metals oxoclusters (TMO) and polyoxometalates (POM), to obtain hybrid materials with enhanced functional (e.g., optical, dielectric, magnetic, catalytic) properties.

## 1. Introduction

When dealing with organic-inorganic hybrid materials, two main underlying concepts are “combination” and “synergy”, since the basic idea behind the development of this rapidly expanding class of materials is to combine organic and inorganic building blocks to afford a material endowed with the properties of both components and eventually to overcome the structural limits of conventional materials (polymers, ceramics, metals, *etc.*) [1–14]. A further aim is to achieve a noticeable improvement of materials properties, since the resulting material typically not only combines the features of both starting systems, *i.e.*, organic and inorganic ones, but can also present additional/enhanced properties deriving from the interaction of both.

The reason why a sharply growing attention is devoted to the development of organic-inorganic hybrid materials, also from the world of production [15,16], is actually the possibility to tailor the properties of the final materials by a careful modification of the chemical nature, structure and amounts of the starting organic and inorganic building blocks. Their mutual distribution and interactions at the organic/inorganic interfaces are further factors affecting the final properties of the materials. The compositional, structural and functional versatility of this peculiar class of materials accounts for their diverse applications ranging from optics and photonics [17–19] to electronics and flexible electronics [20–23], from sensors [24–29] to catalysis [30–34], from electroactive [35,36] or electrochemical [26,37–40] devices, to biomedical [41–44] or to bioactive [45–48] materials. The multifaceted applications of hybrid materials have been recently reviewed by Popall and Sanchez [15,16], whereas the tailoring of hybrid materials to achieve functional properties and combination thereof (*i.e.*, multifunctionality) has been the topic of several contributions and of a dedicated textbook [2,6,8].

In general, organic-inorganic hybrid materials are traditionally defined as a wide, manifold and exciting class of systems which derives from an intimate combination, often mediated by the formation of a chemical bond, of inorganic and organic building blocks [1,2,4,5,9,11,12,14,49–52].

This broad and quite loose definition encompasses several kinds of materials whose common factor is the co-presence of both organic and inorganic components, such as (i) polymers embedding inorganic building blocks [5,53–55]; (ii) inorganic or hybrid (sol-gel) matrices incorporating organic molecules, macromolecules or dyes; (iii) Metal Organic Framework (MOF) [56–67] based on inorganic building block or metal ions connected by polytopic organic ligands; (iv) coordination polymers [68–71] and complexes; (v) nanoparticles and surfaces decorated with organic molecules or macromolecules [72] and (vi) organic-inorganic interfaces and interphases. When seeking for a more stringent classification, and trying to focus on a particular typology of hybrids, we can for instance limit ourselves to materials in which a *host* incorporates a *guest* of different nature and then single out two main classes of systems: (1) either inorganic building blocks (BB) (clusters, nanoparticles, fibers, whiskers, lamellae, *etc.*) are incorporated into a macromolecular polymer backbone (inorganic *guest* in organic *host*) or, *vice versa*; (2) organic molecules or macromolecules (dyes, biomolecules, oligomers or polymers) can be embedded into an inorganic (e.g., silica) matrix (organic *guest* in inorganic *host*).

If we further focus on the first group, *i.e.*, macromolecular networks embedding inorganic components, we enter the manifold world of composite materials, in which the properties of polymers are improved, typically in terms of thermal and mechanical stability, flame retardancy, barrier properties, *etc*., by the incorporation of tailored inorganic fillers. In traditional composites and nanocomposites [11,12,73,74] this combination is addressed by a simple physical mixing or blending of two or more components (“Class I” hybrid materials), based on weak interactions such as van der Waals interactions or hydrogen bonds, although advances have been made to modify filler surface, so to improve the compatibility among the components [75].

At variance to that, in the class of hybrid materials we are more interested in, the so called “Class II” hybrid materials [1,9,14], the stable (typically covalent) anchoring of the guest to the host matrix ensures greater stability and improved performances of the final hybrid material. In fact, the lack of a strong chemical bond generally leads to poor mechanical properties, migration and/or leaching of the guest components within/from the host matrix, phase agglomeration, demixing, which are all detrimental for materials properties.

Whereas the structural (*i.e.*, mechanical, thermal, rheological, *etc.*) properties of these materials are typically ruled by the nature of the organic/inorganic polymer host network and the functional properties are typically related to the nature of the interphase between the two domains or to the chemical nature of the incorporated unit, and these hybrid materials can be endowed, *inter alia*, with interesting optical/photonic, magnetic, electric/dielectric/piezoelectric, electrochemical, catalytic, sensing properties or with bioactivity [2,48].

Different synthetic approaches to hybrid (host/guest) materials have been systematically described by Kickelbick [1,53], Sanchez [76–82] and Schubert [5,54,55] and further authors, and involves either (i) the use of sol-gel process or (ii) the formation of organic polymers in the presence of preformed inorganic components or (iii) the simultaneous formation of both networks. In particular, Kickelbick [53] has described in his review paper the main synthetic approaches to incorporation of a wide variety of inorganic BB into polymer, among which:

(1) Metals or metal complexes, by coordination interactions;
(2) Incorporation of unmodified particles;
(3) *In situ* growth of inorganic particles within a polymer matrix;
(4) Surface modification of clusters and oxoclusters with polymerizable groups;
(5) Surface modification of clusters and oxoclusters with polymerization initiating groups.

As mentioned in the Abstract, among the different inorganic components, which can be added to a polymer to get its hybridization, the resort to structurally defined inorganic building blocks appears as a particularly convenient and viable route to better control their dispersion in the hybrid.

In fact, several advantages of well defined molecular or polynuclear complexes such as clusters, polyoxoanions and oxoclusters with respect to nanoparticles, clays or similar less defined systems can be envisaged [55]:

(1) Clusters are molecules: each cluster in a macroscopic sample has the same composition, size and shape;
(2) They can be synthesized, purified and functionalized by using standard methods of inorganic and organic chemistry;
(3) They can be typically dissolved in common solvents and be easily analyzed by conventional spectroscopic methods;
(4) They are generally crystalline, *i.e.*, their structural determination can be afforded by X-ray diffraction, providing an unambiguous determination of their stoichiometry.

In this context, different authors (see also the relevant literature collected in the given references) reported on the use of (i) polyhedral silsesesquioxanes (POSS) [1,83–91]; (ii) polyoxometalates (POM) [92–97], oxoclusters [5,55,98–100], as inorganic components displaying well defined structures, which can be embedded into polymer networks to afford functional hybrid materials for different applications. By using this general approach, not only bulk hybrid materials could be obtained, but also porous and mesoporous structures, as well as thin/thick hybrid coatings and films[16,101–114].

POSS, having the general formulae R_n_Si_n_O_3n/2_ or spherosilicates (OR)_n_Si_n_O_3n/2_ (with n typically 8) are well known and their applications as fillers in composites and hybrids have already been established. However, they will not be further reviewed in this paper.

POM [94–96,115–117] and oxoclusters are fascinating inorganic systems displaying high nuclearities (number of metal atoms) and their synthesis, main features and applications will be reviewed in the next sections.

To crosslink a polymer by using an inorganic unit, a mandatory requirement to be met is the presence of functional groups, typically reactive or polymerizable ones, on the surface of the inorganic building unit. To achieve it; (i) the synthesis of the cluster in the presence of the functional moiety or (ii) the post-functionalization (by either replacement of a former ligand by the functional one or by functionalization of the ligand itself) are viable routes [55], the former being easier, since the latter requires at least one additional synthetic step and might involve a rearrangement of the structure [118].

The concept of using polyfunctional units to achieve crosslinking is already extensively established in polymer chemistry, and allows to tailor, according to the number and spatial distribution of functional moieties involved in the polymerization, the degree of crosslinking and to obtain either branched or highly crosslinked materials. The same concept can be extended and implemented to hybrid materials, the multi-functionalized inorganic building block playing in this context the role of crosslinker. The benefit over all-organic crosslinkers is represented by the inorganic nature of the building block, possibly generating further improvements in the resulting material, or endowing it with new properties.

The routes for the derivatization of both oxoclusters and POM have been already reported and described by other authors [5,93,119–124]. Typically, the functionalized inorganic building block is directly added to the monomer formulation, before starting the polymerization. The number, arrangement and density of the surface functional groups affect the final crosslinking density of the resulting polymer, which in turn can improve the mechanical and/or thermal properties of the hybrid [4,5,98,125,126].

Several parameters can be changed to tailor the features of the final material: beside the nature and number of metal ions, the polyhedral structure and connectivity of the inorganic building block, further diversity can be generated by varying the chemical nature of the surface functionalization. However, this latter is dictated on the chemical nature of the polymer host, since the chemical bond between organic and inorganic domains is formed among the surface moiety of the inorganic unit and the monomer leading to the formation of the macromolecular skeleton. In this context, several different functional groups (methacrylate, acrylate, norbornene, thiol groups, *etc*.), and polymerization routes (free radical polymerization, cationic photopolymerization [108,127–131], atom transfer radical polymerization (ATRP), *etc.*) have been used to prepare this kind of inorganics-reinforced polymers [132–137].

Upon embedding these inorganic units into a polymer, three main structural issues to be considered are: (i) the integrity of the inorganic cluster; (ii) its distribution/dispersion into the organic backbone and (iii) the connectivity with the polymer segments [55]. As far as the former issue is concerned, being the resulting hybrids typically amorphous materials, *i.e.*, they lack a long-range order, an analytical methods allowing the investigation of short-range order is required.

In the case of the more robust POM, the assumption of stability after incorporation typically holds, and FT-IR and/or solid state NMR can usually confirm this. For less stable BB such as oxoclusters, a powerful tool to assess whether the cluster has retained or not its original structure is X-ray absorption spectroscopy, whose application to the study of organic-inorganic hybrid materials has been reviewed elsewhere [138]. In addition, as far as dispersion into the organic backbone is concerned, being the electron density contrast between the organic and inorganic components large, a very suitable method to study cluster-distribution in the polymer is Small Angle X-ray Scattering (SAXS), whose applications to hybrid materials were discussed elsewhere [139–141]. Finally, the investigation of the nature of the organic/inorganic interphase is very challenging from an analytical point of view, and typically it is addressed by a combination of different characterization methods, such as FT-IR, Raman, solid-state NMR [142–146], *etc*.

## 2. Oxocluster-Based Hybrid Materials

Oxoclusters [4,5,54,55,98,147–150] of early transition metals, otherwise referred by some author as “metal oxide clusters” [4,54,149] are a class of polynuclear compounds, typically based on 3–5 groups metal atoms in their highest oxidation state, such as Ti^IV^, Zr^IV^, Hf^IV^, or Nb^V^ linked by oxygen bridges and coordinated by organic ligands. In some cases, as extensively reported in the following, later transition metals (e.g., Ag) or alkaline earth (e.g., Ba, Mg) metals can also be present in the structure [151]. Oxoclusters display different nuclearities, *i.e.*, number of metal atoms (*n* = 2–12), coordination number of the metal atoms and connectivity fashions (corner, edge or face sharing) of the metal-oxygen coordination polyhedra.

Unlike polyoxometalates (*vide infra*), these compounds are globally neutral and discrete species having the general formula M_y_O_x_(OR)_w_R′_z_, with (R = H or organic group, R′ = organic groups). In most case the bidentate ligand is a carboxylate, and the formula can be written as M_y_O_x_(OR)_w_(OOCR′)_z_ (with R = H or organic group, R′ = organic groups).

Their synthesis typically relies on a controlled hydrolysis of metal alkoxide in the presence of bidentate ligands (typically carboxylic acids) [152]. A schematic description of the different reaction steps, in the case of carboxylate-based oxoclusters is sketched below [152]:

M(OR)j+ k R'COOH⇔M(OOCR')k(OR)j−k+k ROH (1)

ROH+R'COOH⇔R'COOR+H2O (2)

m  M(OOCR')k(OR)j−k+n H2O⇔Mm(OH)2n−[m×(j−k)]On −{2n−[m×(j−k)]}(OOCR')k×m+m×(j−k)ROH (3)

and the reaction path has been elucidated by Kickelbick and coworkers by a combined use of different analytical methods, even in time-resolved fashion.

The key step of the overall reaction is the second one: upon esterification of the partially substituted metal alkoxide in the presence of an excess of carboxylic acid, a stoichiometric amount of water is released in a controlled way in the reaction environment. This controlled amount of water triggers hydrolysis/condensation reactions to form the μ-oxo M-O-M moieties, which are the common structural feature of all these oxoclusters.

For the sake of completeness, it should be highlighted here that, while the above reported mechanism holds for oxoclusters with carboxylate ligands, a modified mechanism occurs for oxoclusters bearing other bidentate ligands (e.g., β-diketonate) or with only alkoxy ligands (for instance Ti_16_O_16_(OR)_32_ or Ti_12_O_16_(OR)_16_). Furthermore, although the proposed scheme takes only into account the explicit generation of water through the esterification step (2), other two reactions could in principle occur (which however have not yet been, to the best of our knowledge, analytically detected in this context), leading respectively (i) to the possible direct formation of M–OH moieties through the reaction of the alkoxide with the carboxylic acid:

M(OR)j+R'COOH→(OR)j−1–M–OH+R'COOR (4)

and (ii) to the direct formation of oxo-bridges through the reaction

M(OR)j+M–OOCR'→M–O–M(OR)j−n+R'COOR (5)

Starting from the pioneering works of Schubert and Sanchez, [54,74,147,148,153–155] these oxoclusters have been extensively used since years as building blocks for inorganic-organic hybrid materials [4,5,54,55,147]. More recently, they have been used as molecular precursors for controlled nucleation of nanostructured oxides [156] or, by exploiting their twelve-fold functionality, as secondary building units (SBU) for Metal Organic Frameworks (MOFs) [157].

Among the first reported examples of these oxoclusters, one of the earliest structurally characterized is Ti_6_O_4_(OR)_8_(OOCMe)_8_ [100,121], obtained by reaction of Ti(OR)_4_ (R = alkyl groups) with acetic acid. In the last years, the synthesis of different early transition metal and main/transition group metal polynuclear complexes (with O–M–O moieties) were extensively explored, resulting in oxoclusters based on Zr [158–161], Hf [162], Ti and Ti-Zr [139,158,163], Ag-Zr [164], Y, Ti-Hf [162], Zr-Ti-Hf [162], Ba [151], Ba-Ti [151], Ti-Pb and Ti-Sr [165], Ti-Y [166], Nb [167], Sn [120,168–176]. Since the 1990s, Hubert-Pfalzgraf *et al.* [167,177–180] have paved the way to this development by exploring original routes for the synthesis of a plethora of mono- and polymetallic (e.g., Pb-Zr, Pb-Ti, Cu-Y, lanthanides, La-Zn, Ba-Ce, and others) mixed alkoxides and oxoclusters as potential single-source precursors for the corresponding mixed functional oxides (e.g., perovskites). Further examples of metal oxide clusters based on other transition elements (Fe, Cr) were obtained by reacting the corresponding metal salts with unsaturated carboxylic acids or carboxylate salts, such as [Fe_3_O(μ-OOCR)_6_L_3_]X (where OOCR = acrylate or 2-butenate and X = counter ion), featuring a triangular M_3_O core [181].

**Figure 1.**
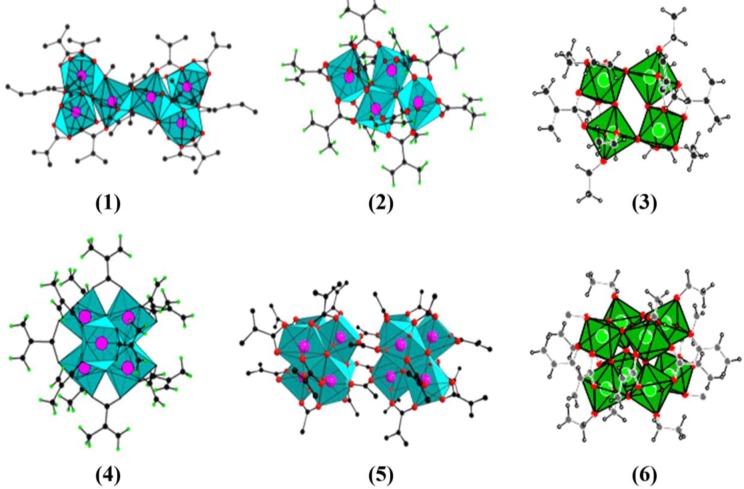
Polymerizable methacrylate (OMc) or acrylate (OAcr)-functionalized transition metal oxoclusters: (**1**) Zr_6_O_2_(OBu)_10_(OMc)_10_ [182]; (**2**) Zr_4_O_2_(OMc)_12_ [161]; (**3**) Ta_4_O_4_(OEt)_8_(OMc)_4_ [183]; (**4**) Zr_6_(OH)_4_O_4_(OMc)_12_ [160]; (**5**) [Zr_6_(OH)_4_O_4_(OOR)_12_]_2_ [184]; (**6**) Ta_8_O_12_(OEt)_8_(OAcr)_8_.

The chemical properties, the tailored synthesis and modification, the structural issues of these oxoclusters, as well as their use as building blocks for the preparation of hybrid materials were thoroughly described in some reviews and research papers, already cited in the Introduction. Selected examples of these oxoclusters are depicted in Figure 1.

Bidentate ligands bearing polymerizable moieties may absolve an important function: by symmetrically functionalizing the oxocluster with functional groups (more typically acrylates or methacrylates), they trigger and enable the crosslinking of the resulting polymer network which is formed upon reaction with suitable monomers in a further synthetic step.

Coming to the topic of this paper, and concerning the above mentioned nature of the functionalization, in the literature are reported, *inter alia*, oxoclusters which have been functionalized with acrylates or methacrylates [158–164,182,185–187] for free radical polymerization, norbornene-2-carboxylate derivatives for ring-opening metathesis polymerization [125], 4-pentynoate derivatives for click reactions [188], 2-bromo-isobutyrate derivatives as initiator for atom-transfer radical polymerization [189], and thiol carboxylate-substituted metal oxocluster for thiol-ene polymerization [142,190].

In general, the incorporation of an oxocluster in a polymer matrix through the formation of stable covalent bonds induces strong changes in the materials properties, as extensively outlined in previous works [4,5,98,126,148,149]. The changes of some materials properties include:

- Linear polymers such polymethylmethacrylate (PMMA) or polystyrene (PS) are turned into crosslinked polymers: the polymers are no longer soluble in organic solvents, but swell instead, as expected for crosslinked polymers; the crosslinking typically increases with the oxocluster amount;
- Improvement of the thermal stability with respect to the neat polymers;
- Improvement of the mechanical properties (strength, hardness, brittleness, scratch resistance, *etc.*), as a further consequence of the crosslinking;
- Enhancement of the dielectric properties (e.g., lowering of ε and tan δ);
- Improved chemical and photochemical stability.

Taking into account such considerations, these oxoclusters have been extensively used for the synthesis of hybrid materials, both for structural and functional issues, but in this contribution we will focus only on the latter. For the former, the interested reader can refer to references [4,5,54,55,98,111,126,140,147,191–193].

It is worth to highlight that the functional properties of the final oxocluster-reinforced hybrid material can be either related to some inherent property of the oxocluster itself, which is transferred to the material, or to some functional effect which is determined by the presence of the oxocluster into the polymer backbone, for instance due to chain dynamics modulation or to the formation of voids in the chain packing. In the following, selected examples on the possibility to endow the final hybrid material with functional properties are discussed.

A first case in which a functional property of the oxocluster is *tout court* transferred to the resulting hybrid material concerns magnetic oxoclusters-based hybrids described by Schubert *et al.* [194]. In this work, the authors embedded the Mn oxocluster Mn_12_O_12_(OAcr)_16_ in polyacrylate matrix and then investigated, *inter alia*, the magnetic properties of the resulting hybrid materials which were proven to be the same as the isolated oxocluster. A very similar study was also carried out by Willemin *et al.*, leading to analog results [195]. These two studies showed that the polymerization of the magnetic clusters in the presence of organic monomers allows the preparation of magnetic materials that can be processed like typical organic polymers but retain the properties of the embedded molecular magnets. The Mn_12_ clusters of general composition Mn_12_O_12_(OOCR)_16_ are in fact well know and prototypal examples of magnetic molecular clusters. The superparamagnetic properties reported by Sessoli *et al.* [196–199] in these Mn oxoclusters, due to the slow relaxation of the magnetization, have disclosed the possibility to use them for storing information at the molecular level.

Always concerning magnetic oxoclusters, a proposed approach relies on the use of miniemulsion to prepare magnetic oxoclusters based hybrid materials. In particular, either a manganese-oxo or manganese-iron-oxo cluster Mn_12_O_12_(VBA)_16_(H_2_O)_4_ and Mn_8_Fe_4_O_12_(VBA)_16_(H_2_O)_4_ (where VBA = 4-vinylbenzoate), were prepared and characterized. Polymerization of the functionalized metal oxoclusters with styrene, under miniemulsion conditions [200,201], produced monodispersed polymer nanoparticles endowed with magnetic properties for potential magnetic imaging applications [202,203].

In the field of optical applications, few examples are reported, [79,165,204]. As far as the refractive index is concerned, an increase of the refractive index of the polymer matrix upon embedding of heavier metal (Zr, Hf) oxoclusters would be expected. Actually, since the amount of oxocluster embedded in the polymer network is generally low (1 at%–3 at%), no relevant change is expected in the variation of optical properties, such as, for example, transparency. In this regard, an example of cluster-reinforced hybrid material concerned with tuning of optical properties is based on the addition of metal oxoclusters, into an inorganic-organic hybrid host material (Organically Modified Ceramics, OrMoCer) [174]. In particular, different benzoic acid functionalized titanium oxo-alkoxo-clusters Ti_6_O_4_(C_6_H_5_COO)_8_(OR)_8_ (R = Et, *^n^*Pr-, *^n^*Bu-) were prepared and their dispersion in Ormocer matrix led to a homogeneous, photo-patternable hybrid system with tailored properties. The refractive index of the host Ormocer matrix could be increased from 1.552 to 1.575 at 635 nm by the addition of these titanium clusters (*ca.* 2.2 mol%). The novel hybrid materials displayed similar flexibility in processing than the host matrices, offering a broad variety of applications in microsystems technology.

Instead, among optical properties induced by the nature of the oxocluster, it should be mentioned the photochromicity [205–208] observed by Sanchez and coworkers in hybrid materials produced by the embedding of the Ti_16_ oxocluster into poly(hydroxyethylmethacrylate). The resulting hybrid materials become dark blue upon UV-Visible irradiation, and this effect was ascribed to the absorption created by the intervalence band associated with the photogeneration of localized titanium(III) polarons. The presence of mixed-valence Ti(III)–Ti(IV) entities was shown through UV-Visible and EPR measurements (ESI). This photochromic behavior is reversible in the presence of oxygen which yields the back oxidation of the Ti(III) centers into Ti(IV).

As a further example, transparent di-ureasil hybrids containing a methacrylic acid-modified zirconium tetrapropoxide (ZrMcOH) clusters and incorporating EuCl_3_ and [Eu(tta)_3_(H_2_O)_2_] (tta = thenoyltrifluoroacetonate) complex were proven to be multi-wavelength emitters for applications in optics [165,204].

As far as the electric properties are concerned, a wide applications of oxocluster-reinforced polymers has been assessed in the field of dielectric films, for instance for the development of Field Effect Transistors (FET) and dielectric materials for electronic devices [158,183,209–212]. In general, in the materials prepared by embedding of the oxocluster into PMMA, a lowering of the dielectric constant and of tanδ could be evidenced. In this case, these functional properties of the material, characterized by broad band dielectric spectroscopy, could be traced back to modulation of the chain dynamics, induced by the presence of the inorganic symmetrically functionalized crosslinker, and to the creation of voids in the polymer matrix, induced by the presence of the oxocluster itself, which does not migrate in the materials. Accordingly, the electrical properties of the hybrid materials were proven to be strongly affected by the molar ratio between cluster and monomer.

A further example of improvement of functional properties, and ascribable to the presence of the oxocluster, is the fairly good barrier properties against corrosion evidenced for a polyacrylate matrix embedding the Zr_4_ oxocluster [213]. In this regard, electrochemical impedance spectroscopy (EIS) was performed in order to evaluate if the coatings actually protect the metallic substrate from corrosion. Although the water uptake of hybrids is greater than that of pure PMMA and some improvements in the process are required, the hybrid coatings appear promising as barrier against corrosion and they generally behave better than pure PMMA, when deposited on different aluminium alloy substrates.

Oxoclusters-based hybrids, typically based either on polymethacrylate or on hybrid silica as host matrix, have been successfully used also for the development of different protective coatings for wood and cellulose [111,114,214].

The use of oxoclusters-based hybrids has been very recently extended to materials for catalytic applications. Actually, the idea of protecting a catalytically active system by embedding it in/anchoring it on a matrix is the underlying and widely used concept in heterogeneous catalysis. The use of hybrid materials to “heterogenize” catalysts, in particular by the embedding of the catalyst into a polymer matrix, is a fertile field of research. Recently, we have evidenced the possibility to use zirconium-based oxoclusters to activate hydrogen peroxide, for the oxidation of organic substrates [215]. The oxidation of methyl *p*-tolylsulfide to the corresponding sulfoxide and sulfone was chosen as model reaction, showing an interesting selectivity towards the oxidation of the sulfoxide. Now, we have embedded Zr and Hf oxoclusters into PMMA matrix and proved their effectiveness towards the oxydesulfurization of a model fuel [216]. To this aim, we have exploited such reactivity to perform the oxidation of dibenzothiophene (DBT) to the corresponding sulfoxide (DBTO) and sulfone (DBTO_2_). Up to 90% yield for DBT conversion was obtained in 24 h, with an 85% selectivity for DBTO_2_. In most cases, thanks to the enhanced affinity of the polymeric matrix towards polar substrates and solvents, the heterogeneous set-up have shown to be more efficient than the corresponding homogeneous systems, and has allowed the recovery and recycling of the catalytic species. FT-IR, SS-NMR and XAS showed good stability of the hybrids under catalytic conditions.

## 3. Polyoxometalate-Based Hybrid Materials

Polyoxometalates (POM) discovery dates back to the last third of the XIX century, when early transition elements of groups V and VI, such as Nb, V, Ta, Mo, and W in their higher oxidation states (configuration d^0^ or d^1^) were found to form polynuclear oxoanions in aqueous solution at acidic pH. Under such conditions, they can form molecular compounds of variable dimensions, ranging from few Ångström to tens of nanometers [95–97,116,117]. A general classification is based on their composition, essentially represented by two types of formula: [M_m_O_y_]^p−^ and [X_x_M_m_O_y_]^q−^ where M is the main transition metal constituent of the polyoxometalate, O is the oxygen atom and X can be a non-metal atom as P, Si, As, Sb, another element of the p block, or a different transition metal. The first formula refers to *isopolyanions*; the second one refers to *heteropolyanions*. When the latter incorporate different transition metals, they are called *Transition Metals Substituted Polyoxometalates* (TMSP). In most cases, the structure of the POMs derives from the aggregation of octahedral units MO_6_, although MO_4_ tetrahedra can also be present. Oxygen atoms exhibiting simple bonds with the metal allow the condensation between two octahedral units, with the formation of μ-oxo bridges between two metals ions. The octahedra can thus be condensed in three different ways: (i) corner sharing; (ii) edge sharing; (iii) face sharing. One oxygen atom—or maximum two-show a double bond character with the central M atom and they are not shared with other M atoms. These terminal oxygen atoms are essential for the aggregation to take place into discrete structures and not in an extended material (as for most common main-group element oxides: silicates, phosphates, germanates, *etc*.) [97].

**Figure 2.**
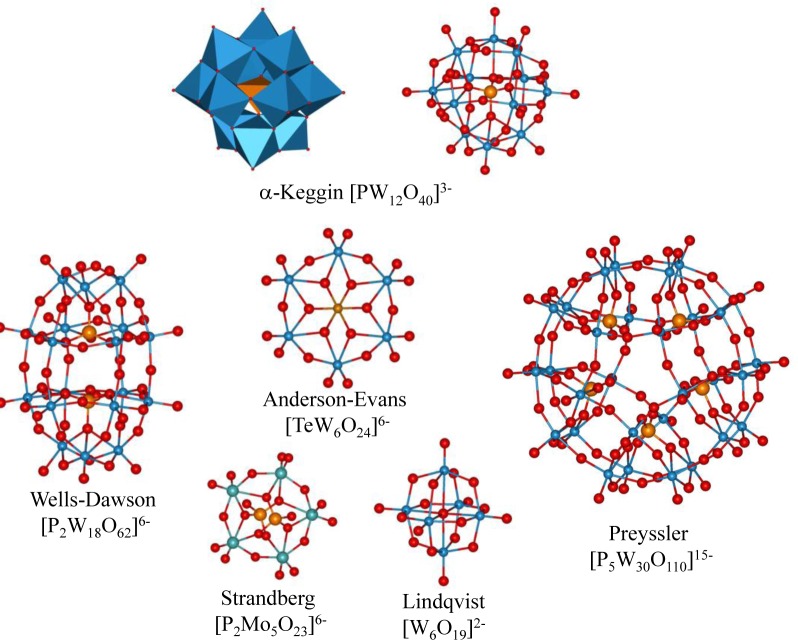
Top: polyhedra (left) and ball & stick (right) representations of α-Keggin polyoxotungstate [PW_12_O_40_]^3−^; bottom: ball & stick structures of other representative polyoxometalates.

Among the most important classes of polyoxometalates there are the Keggin heteropolyanions. Their general formula is: [XM_12_O_40_]^n−^, with M = Mo (VI) or W (VI). In 1934 Keggin obtained the structure of the hexahydrated dodecatungstophosphoric acid by powder X-ray investigation [217]. This structure is called α-Keggin and consists of a central PO_4_ tetrahedron surrounded by 12 octahedra WO_6_ belonging to the mono-oxo terminal type. These octahedra arrange themselves in four triplets M_3_O_13_ where the three octahedral units aggregate by edge-sharing. Finally, the four different triplets condense each other by corner-sharing (Figure 2). Structural isomers of Keggin polyanion are formally obtained from the α structure by 60° rotation of one (β isomer), two (γ isomer), three (δ isomer) or four (ε isomer) triplets M_3_O_13_. Such isomers are characterized by lower symmetry and by a decreased thermodynamic stability with respect to the α structure.

Beside Keggin polyanions, a great variety of structures (Wells-Dawson [X_2_M_18_O_62_]^n−^, Anderson-Evans [XMo_6_O_24_]^n−^, Lindqvist [M_6_O_19_]^n−^, Strandberg [P_2_Mo_5_O_23_]^6−^, Preyssler [P_5_W_30_O_110_]^15−^, *etc*.) can be obtained in particular synthetic conditions by tuning some specific parameters like concentration, stoichiometric ratio between the reagents, temperature, and pH [218].

Since POMs are hydrolytically instable in alkaline media, it is possible to exploit this behavior to promote in a controlled way the selective formation of structural defects. The resulting vacant or “lacunary” POM complexes [117,219] derive from the saturated precursors through the formal loss of one or more MO_6_ octahedral units and can be used as ligands for transition metals and organometallic groups or as building blocks for the preparation of oligomeric POM aggregates.

What is noteworthy is the possibility for isostructural polyoxometalates to show different properties depending on the heteroatoms [220,221]. In addition, the choice of a suitable counterion for such complexes is fundamental to allow their solubilization in a wide range of solvents: from apolar ones (toluene, dichloromethane), by using lipophilic cations such as dimethyloctadecy ammonium (DODA), to water, with alkaline counterions or protons.

Different scientific fields, such as medicine, materials science, and catalysis, are particularly interested in the properties of polyoxometalates for their acidity, redox activity, thermal and oxidative stability, high charge density, electron acceptor/storing capability and magnetism [222–226]. In particular, polyoxometalates are more stable towards the oxidative degradation than generic organic molecules, since they are made of metals in their higher oxidation state [227,228]. Therefore they can be successfully exploited for the activation of sustainable oxidants like dioxygen and hydrogen peroxide to perform the oxidation of organic substrate [229–231], as well as for advanced application as water splitting [232,233].

Due to their manifold applications, POMs immobilization may reduce cost and environmental impact of POM-based catalysts, while their confinement into different matrixes may be useful to design opto-electronic devices or carrier systems for the delivery of biologically active POMs. The introduction of POMs into processable polymeric matrices may be of interest for the development of heterogeneous catalytic systems to be employed in continuous processes based on fixed-bed reactors and membrane reactors. In addition, the polymeric host can play a role in enhancing reaction selectivity, through differential sorption/permeability of reagents [234,235].

The physical blending method is the simplest way to fabricate POM/polymer hybrid materials. Some water-soluble polymers, such as poly(vinylalcohol) (PVA), poly(ethylene glycol) (PEG), agarose, polyacrylamide and poly(vinyl pyrrolidone) (PVP), were used as compatible matrices for hydrosoluble POMs. Dipping or spin-coating methods were used to obtain films on different solid supports [92], while the electro-spinning technique was used to obtain fibers [236]. In order to match the hydrophobicity of water-insoluble polymers, POMs can be associated with suitable counterions featuring high affinity towards the organic matrix. This was successfully achieved with the decatungstate [W_10_O_32_]^4−^, which was isolated as tetrabutylammonium salt and incorporated within polymeric membranes (polysulfone PS, polyether-etherketon PEEK-WC, polyvinylidene difluoride PVDF, polydimethylsiloxane PDMS) [237], or as fluorophilic salt (with the cation [CF_3_(CF_2_)_7_(CH_2_)_3_]_3_N^+^CH_3_), for the incorporation within the perfluorinated polymer Hyflon^®^ [238]. The resulting materials were used as photocatalysts to oxidize either alcohols or hydrocarbons, respectively, and a matrix effect was observed on reaction selectivity. Within this *scenario*, the preparation of surfactants encapsulated POMs (SEP) is a well-known strategy to obtain POMs with high affinity towards low polarity media. For example, the compatibility between the photoluminescent complex (DODA)_9_(EuW_10_O_36_) and the polystyrene matrix allowed the fabrication of hybrid polymeric films with highly ordered honeycomb-like structures. The films were relatively stable and enabled to study photoluminescence and redox properties of the embedded POM [239].

Owing to the lack of strong chemical interactions between the POMs and the polymer, the low stability of these hybrid materials limits their practical applications. An interesting perspective to increase the composite stability consists in using polymerizable surfactants, such as dodecyl[11-(methacryloyloxy)-undecyl]dimethylammonium bromide (DMDA) and cetyl(2-methacryloyloxyethyl)dimethylammonium bromide (CMDA), in order to establish strong interaction between the counter cation and the polymeric matrix obtained *in situ* [240]. A miniemulsion polymerization was also developed to incorporate POMs into PS latex, providing POM protection towards aqueous environment [241].

Another convenient strategy to effectively disperse polyanions into hybrid materials consists in the exchange of their counterions with positively charged polyelectrolytes (polyallylammonium, poly(diallyldimethylammonium), polyviologens, cationic dendrimers, and dendrons) [242–245], or polymers such as polyethyleneimine, poly(4-vinylpyridine), polyaniline, chitosan in their quaternized/protonated form. In this way, a broad number of hybrid materials and thin films were prepared, including hierarchical layer-by-layer (LbL) structures, with applications as opto-electronic devices, catalysts, sensors and antibacterial surfaces [37,92,246–249].

An even more stable interaction between a POM and a polymer was observed in a ultrathin multilayer film, consisting of Keggin anions [PMo_12_O_40_]^3−^ and of a photosensitive diazo resin. After the initial absorption of the anionic clusters onto the positively charged polymer interface, UV irradiation was employed to foster the photodecomposition of the diazo resin, with loss of N_2_, and the photoreduction of the POM. The enhanced stability against solvent etching was explained by the formation of strong Mo-O-C interactions [250].

The previously described assembly methodology exploits multiple interactions between the polycharged domains, but is only limited to polyelectrolytes. The most appealing alternative is thus offered by the formation of covalent bonds between POMs and the polymeric backbone.

As in the case of transition metal oxoclusters described in the former section, the preparation of covalently-linked hybrids generally requires surface modification of the POMs structure. This can be easily achieved upon covalent decoration of vacant polyanions. The nucleophilic oxygen atoms bordering the surface defect can indeed react with electrophilic reagents to yield organosilyl, organostannyl, organophosphonyl, and organogermyl hybrid derivatives [124,251,252]. The covalent functionalization of vacant polyoxoanions generally imparts a stabilization to the POM unit while tuning its electronic properties. The derivatization of polyoxometalates was exploited to obtain: (i) self-assembled supramolecular aggregates, including photoresponsive nanostructured systems; (ii) amphiphilic POM derivatives with solvophobic behavior in water/air and water/oil interface, polar or nonpolar solvents; (iii) conjugates with chiral and biological molecules, for application in enantioselective catalysis or recognition of biological targets; (iv) POM-supported organometallic catalysts, dendrimers, photosensitizers and sensing units. In addition, suitable organic pendants can foster POM immobilization onto solid supports [93].

The main disadvantages of covalent grafting are related to the modified properties of the functionalized POMs with respect to their precursors, and to the need of applying one or more derivatization steps, usually requiring non-conventional conditions/purification techniques. For these reasons, a low number of suitable POMs are available. Nevertheless, as for the oxoclusters, the incorporation of organic moieties provides various options for an easier processability and following integration into functional architectures and devices. A number of advantages are thus available when applying this approach, including: control of the interaction between the different domains, better dispersion and improved stability of the assembly.

**Figure 3.**
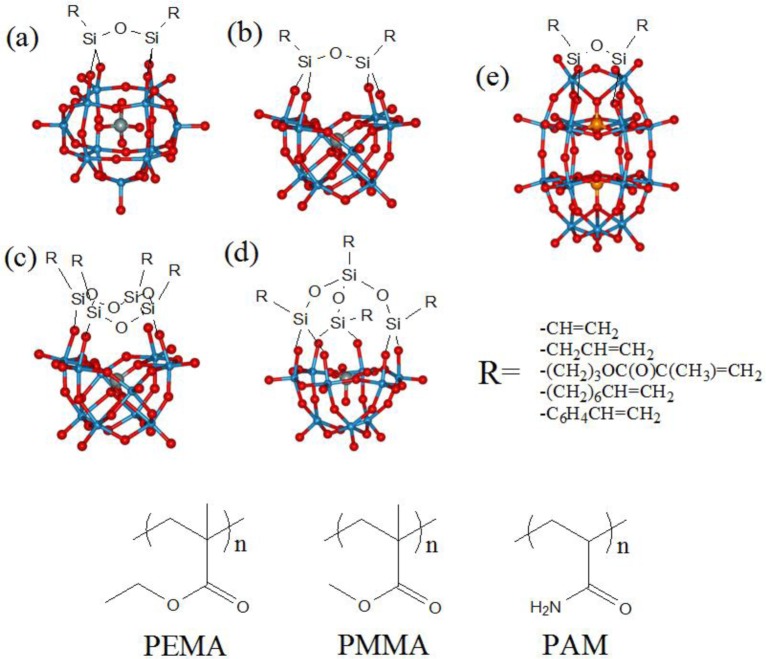
Top: hybrid polyoxotungstates containing polymerizable organosilane pendants: (**a**) bis-(organosilyl) undecatungstosilicate [α-SiW_11_O_39_(RSi)_2_O]^4−^; (**b**) bis- and tetrakis(organosilyl) decatungstosilicates [γ-SiW_10_O_36_(RSi)_2_O]^4−^; and (**c**) [γ-SiW_10_O_36_(RSiO)_4_]^4−^; (**d**) tetrakis(organosilyl) nonatungstosilicate [α-SiW_9_O_34_(RSi)_4_O_3_]^4−^; (**e**) bis-(organosilyl) monovancant Well-Dawson [α_2_-P_2_W_17_O_61_(RSi)_2_O]^6−^. Bottom: acryl-based polymers.

Among hybrid POMs, several polymerizable organosilyl-modified polyoxotungstates (e.g., [α-SiW_11_O_39_(RSi)_2_O]^4−^, [γ-SiW_10_O_36_(RSi)_2_O]^4−^, [γ-SiW_10_O_36_(RSiO)_4_]^4−^, [α-SiW_9_O_34_(RSi)_4_O_3_]^4−^, with R = vinyl(–CH=CH_2_), 3-(methacryloxy)propyl (–(CH_2_)_3_OC(O)C(CH_3_)=CH_2_), octenyl (–(CH_2_)_6_CH=CH_2_), were prepared (Figure 3) [253–255].

In a first example, Judeinstein used trichloro- or triethoxy- organosilanes to introduce the following R groups: vinyl (–CH=CH_2_), allyl (–CH_2_CH=CH_2_), 3-(methacryloxy)propyl (–(CH_2_)_3_OC(O)C(CH_3_)=CH_2_), styryl (–C_6_H_4_CH=CH_2_) groups on the undecatungstate α-Keggin polyanion [α-SiW_11_O_39_]^8−^ (Figure 3a). Hybrid and branched polymers based on such POM units were then synthesized via radical polymerization, in the absence of any additional monomer, to obtain a POM-based polymer. Transparent thin films of these polymers turn reversibly to blue upon UV irradiation or electrochemical reduction, due to formation of W(V) centers, being, thus, promising for the development of photochromic and electrochromic devices [256].

The vinyl-functionalized [α-SiW_11_O_39_(CH_2_=CHSi)_2_O]^4−^ (Figure 3a) was converted into the free acid form and copolymerized with butylacrylate and 1,6-hexanediol diacrylate, under UV irradiation, in the presence of a photoinitiator. The resulting material exhibits proton conductivity of 0.17 S·cm^−1^ at 80 °C, *i.e.*, close to the standard operating conditions of proton-exchange membrane (PEM) fuel cells [257].

Hybrid networks based on the decatungstosilicate Keggin compound [γ-SiW_10_O_36_{CH_2_=C(CH_3_)C(O)O(CH_2_)_3_Si}_2_O]^4−^ (Figure 3b) were obtained upon copolymerization with ethyl methacrylate. To foster further crosslinking, the polymerizable cation CH_2_=C(CH_3_)C(O)O(CH_2_)_2_N^+^(CH_3_)_3_ was associated to the same POM. The inorganic component was shown to give a substantial contribution to the swelling properties of the resulting polymeric gel into polar organic solvents, depending on monomers ratio and cross-linking density [258]. In a parallel investigation, the possibility to obtain hydrogels based on the amphiphilic sodium salt of [γ-SiW_10_O_36_{CH_2_=C(CH_3_)C(O)O(CH_2_)_3_SiO}_4_]^4−^ (Figure 3c), copolymerized with acrylamide, was also demonstrated. In this case, the hybrid material is able to swell in the presence of water, depending on POM concentration and aggregation state, up to 150 g/g [259]. Magnetic nanosized γ-Fe_2_O_3_ particles were then included in the absorbent material. The nanoparticles retain their rotational mobility within the network, and their controlled release upon swelling of the hydrogel was described [260].

The polyanions [SiW*_w_*O*_z_*{CH_2_=CH(CH_2_)_6_Si*_x_*}O*_y_* ]^4−^ with *x* = 2, *w* = 11, *y* = 1, *z* = 39; *x* = 2, *w* = 10, *y* = 1, *z* = 36 and *x* = 4, *w* = 9, *y* = 3, *z* = 34 (Figure 3a,b,d) were copolymerized with methyl methacrylate and ethylene glycol dimethacrylate, in the presence of porogenic alcohols, to form porous materials. The hybrid copolymers were then used in acetonitrile or in a biphasic (*n*-octane/acetonitrile) media, in the presence of aqueous hydrogen peroxide, to catalyze the oxidation of organic sulphides, including dibenzothiopene (DBT). In particular, the hybrid polymer based on the decatungstosilicate polyanion removed sulfur-based compounds from *n*-octane in 90 min, at 60 °C, and reduced the sulfur content to about 10–8 ppm, highlighting the promising potential of the supported POMs in the oxydesulfurization of hydrocarbons [255].

The Dawson polyanion ([α_2_-P_2_W_17_O_61_{CH_2_=C(CH_3_)C(O)O(CH_2_)_3_Si}_2_O]^6−^, Figure 3e) in its free-acid form was polymerized with methyl methacrylate. The acidity of the hybrid copolymer was evaluated in CH_3_CN solution (homogeneous conditions) and in CH_3_OH (heterogeneous conditions) by using Hammett indicators, showing the retention of the original POM acidity and suggesting its use as a promising solid acid catalyst [261].

Other hybrid polyoxotungstates with reactive functional groups, such as thiol, sulfocyanide, amino, are suitable for their covalent immobilization onto supports [262–264]. For example, the Dawson POM [α_2_-P_2_W_17_O_61_{HS(CH_2_)_3_Si}_2_O]^6−^, carrying thiol groups as pendant arms, was reacted via nucleophilic substitution with nanolatex particles obtained by the copolymerization of styrene, vinyl benzyl chloride and hydroxypropyl methacrylate (cosurfactant) in an oil-in-water microemulsion. The stable colloidal particles display a closely packed inorganic shell with photochromic behavior [265].

Another organically modified Dawson POM, [α_2_-P_2_W_17_O_61_(N_3_CH_2_C_6_H_4_Si)_2_O]^6−^, was immobilized on the functionalized channels of a macroporous resin. To this aim, a polystyrene-divinylbenzene matrix containing benzylamine residues were modified by condensation with pentynoic acid, then the bis-azido POM was grafted via click chemistry. The efficiency and the stability of the solid catalyst were demonstrated studying the activation of H_2_O_2_, for the oxidation of tetrahydrothiophene (THT). The catalytic system allowed the quantitative conversion of the substrate to its corresponding sulfoxide in 12 h, at room temperature, in acetonitrile, where it did not show any POM leaching and was thus re-used up to five times [266].

A different approach can be followed when using arylimido derivatives of the Lindqvist hexamolybdate [Mo_6_O_(19−_*_x_*_)_(NAr)*_x_*]^2−^ (with Ar = arly groups). In these hybrids, the organic p electrons may extend their conjugation to the inorganic framework, thus resulting in strong d–p interactions. Organoimido derivatives of POMs with a remote organic functional group were thus successfully used as building blocks to prepare POM–organic hybrids with different architectures [267].

**Figure 4.**
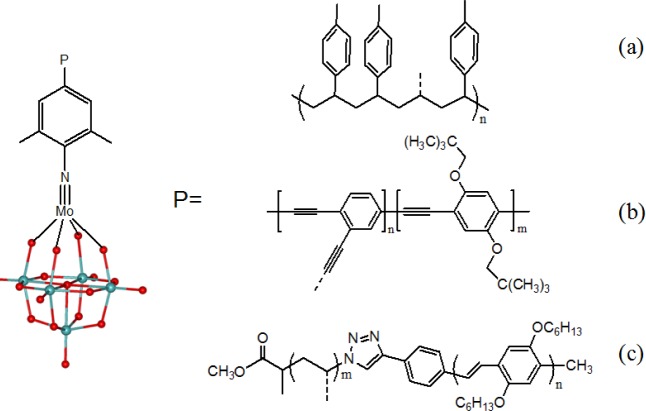
Hybrid polymers based on organoimido-functionalized hexamolybdate as side-chain pendants.

The mono *p*-styrenyl substituent in the derivative [Mo_6_O_18_{NC_6_H_2_(CH_3_)_2_CH=CH_2_}]^2−^ allowed the polyoxometalate complex to be introduced as a pendant group in polystyrene chains, (Figure 4a) [268]. Thanks to the selective reaction of octamolybdate ion [α-Mo_8_O_26_]^4−^ with iodo aryl amine, a *cis* bifunctionalized hexamolybdate [Mo_6_O_17_(NArI)_2_]^2−^ was obtained and polymerized via Pd catalyzed C–C coupling with a diethynylbenzene derivative (2,5-di(2,2-dimethylpropoxy-1,4-diethynylbenzene) [269]. The main chain-POM-containing linear polymer was then employed in photovoltaic devices, whereby a thin layer of polymer was sandwiched between a transparent anode (indium-tin oxide, ITO) and a metal cathode. In this case, the photoinduced charge separation led to a power conversion efficiency of 0.15%. Although this value compares well with other conjugated polymers, the low efficiency was explained in terms of the poor charge transporting properties of the polymer [270]. In a following paper, the authors described the preparation of a π-conjugated polymer, based on poly(phenylene ethynylene), bearing imido-functionalized hexamolybdates as side-chain pendants (Figure 4b). They observed a polymeric backbone fluorescence quenching and suggested a through-bond photoinduced electron transfer as the dominant mechanism [271]. Even better achievements were envisaged for a diblock copolymer containing an oligo(phenylene vinylene) (OPV) rigid block and a polystyryl-type (PS) flexible block with hexamolybdate pendants. In this case, a click chemistry step was used to join together the OPV block and the PS block, while the hexamolybdate was added in a post functionalization step, with formation of imido bonds (Figure 4c). The POM cluster in a solid film was found to quench 74% of the OPV fluorescence [272].

As a final approach it is worth to mention the possibility of preparing bi-functional materials through the condensation of bis derivatized POMs with different functional molecules, such as porphyrins. Hybrid polyoxometalate-porphyrin copolymeric films were obtained by the electro-oxidation of zinc octaethylporphyrins in the presence of the Anderson hexamolybdate [MnMo_6_O_18_{(OCH_2_)_3_CNHCO(4-C_5_H_4_N)}_2_]^3−^. These films were applied for the photocatalytic reduction of Ag^I^_2_SO_4_ in the presence of propan-2-ol [273].

## 4. Conclusions

An overview on the research results on the preparation and properties of oxoclusters/POMs polymeric hybrid materials has been herein described and discussed. Different methodologies for the preparation of such composite materials have been presented, focusing our attention on the most stable and promising covalent strategies.

As a first remark, incorporating oxoclusters/POMs into suitable polymer matrices may increase material processability and long-term stability, as well as thermomechanical properties. Furthermore, and more importantly, the possibility to chemically tailor the composition, structure and functionalities of the inorganic building block and of the polymer matrix pave the way to the obtainment of multifunctional materials. To address this stimulating challenging, the role of synthetic chemistry, encompasses not only the preparation of new inorganic BB with functional properties, such as redox, luminescence or magnetic properties, but also their careful decoration with functionalities to match them with suitable organic counterparts providing better compatibility, stability and performances.

Indeed, we believe that such materials deserve much more attentions, and different combinations of monomers and inorganic building blocks will offer new possibility to strength most of the preliminary but promising studies on electro/photo active or catalytic materials.

A further exciting perspective could be the combination, also aimed at multifunctionality, of POM and oxoclusters to enhance the functional properties of the inorganic counterpart of the hybrid materials.
